# Response to considerations regarding Maximal Lactate Steady State determination before redefining the gold‐standard

**DOI:** 10.14814/phy2.14292

**Published:** 2019-11-23

**Authors:** Andrew M. Jones, Mark Burnley, Matthew I. Black, David C. Poole, Anni Vanhatalo

**Affiliations:** ^1^ Sport and Health Sciences University of Exeter St. Luke's Campus Exeter UK; ^2^ School of Sport and Exercise Sciences University of Kent Medway UK; ^3^ Department of Kinesiology Kansas State University Manhattan Kansas

## Abstract

We reinforce the key messages in our earlier review paper that critical power, rather than maximal lactate steady state, provides the better index for defining steady‐state vs non‐steady state physiological behaviour during exercise.

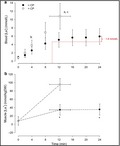

We thank Drs Garcia‐Tabar and Gorostiaga for their interest in our work. We appreciate some of their commentary on the historical development of the lactate threshold (LT) concept, despite the lack of relevance to the key arguments made in our paper (Jones, Burnley, Black, Poole, & Vanhatalo, 2019). As long‐term scholars in this field, we are well aware of the early papers by pioneers in exercise physiology such as Bang and Owles, but it does no harm for a wider readership to be reminded of them.

We agree with Drs Garcia‐Tabar and Gorostiaga that several less tedious methods are available to estimate the maximal lactate steady state (MLSS) and that MLSS is related to (but higher than) LT (e.g. Jones & Doust, 1998). We also agree that poor reliability of blood [lactate] measurement is a major limitation in this field, with it being unwise to presume that errors in blood [lactate] are either unidirectional or predictable. We would emphasize, however, that this problem is *not* overcome, but is rather exacerbated, when the change of blood [lactate] during exercise (e.g. between 10 and 30 min) is considered because, in this situation, MLSS is defined by two measurements that are potentially erroneous. But, even if it were possible to estimate MLSS with greater accuracy, it does not follow that MLSS should be considered the gold‐standard index of endurance capacity ‐ a point that appears to have been missed by our correspondents.

Indeed, none of the points made by Drs Garcia‐Tabar and Gorostiaga challenge the fundamental message in our paper that MLSS (based on the one single, and flawed, metabolic biomarker of blood [lactate]) will underestimate the true maximal metabolic steady state as given by critical power (CP), which has been shown repeatedly to clearly partition steady state from nonsteady state behavior in skeletal muscle metabolic, blood acid‐base and pulmonary O_2_ uptake responses to exercise (Poole, Burnley, Vanhatalo, Rossiter, & Jones, 2016). This is, in part, related to poorly justified criteria for MLSS assessment, namely an increase in blood [lactate] of greater than 1 mM between 10 and 30 min of exercise.

To further illustrate this point, in Figure 1 we have re‐presented data from a recent study in which we measured both skeletal muscle [lactate] and blood [lactate] during continuous exercise performed just below and just above CP (Vanhatalo et al., 2016). It is clear that muscle [lactate] is stable over the latter part of the <CP exercise bout. However, blood [lactate] rises by 1.8 mM between ~10 and 24 min, indicating that the power output is higher than MLSS using the conventional criteria and definition. This figure therefore underlines that: (a) CP is the appropriate metric when the goal is to identify the boundary between the heavy and severe exercise intensity domains; and (b) MLSS underestimates the actual muscle metabolic steady state.

**Figure 1:**
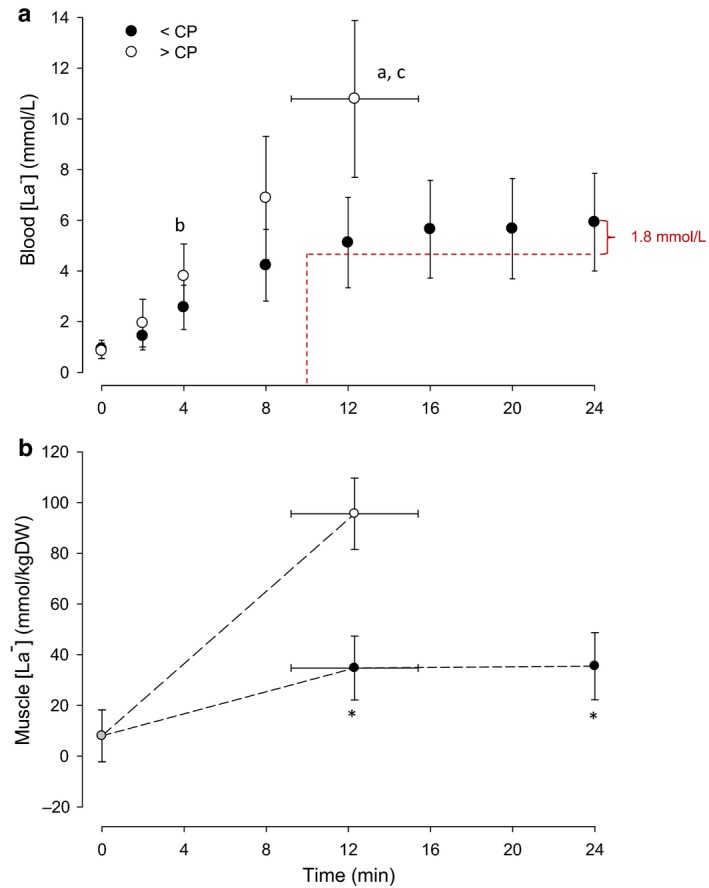
Group mean blood lactate ([La‐], a) and muscle lactate ([La‐], b) responses to exercise below (black symbols) and above (white symbols) the critical power (redrawn from Vanhatalo et al., 2016). (a) Different from end‐exercise value during the <CP test (*p* < .05); (b) Different from the same time point in the < CP test (*p* < .05); (c) Different from the value at 12 min in the <CP test (*p* < .05); *Different from the end‐exercise value during the >CP test (*p* < .05)

Drs Garcia‐Tabar and Gorostiaga argue that an advantage of MLSS assessment is that it permits identification of heart rate zones for training prescription but they apparently fail to appreciate that the same is true for CP! Indeed, a central theme in our paper is that CP is preferable to MLSS because it permits a more accurate estimate of the boundary separating the heavy and severe exercise intensity domains which produce discrete physiological response profiles relevant to fatigue development, exercise prescription and adaptations to training. Finally, the suggestion that tests requiring volitional exhaustion are unfeasible or undesirable in elite athletes is patently untrue but, in any case, it should be pointed out that power‐duration curves can be constituted from training records or race performances.

The selection of exercise tests should be based on rigorous science as well as convenience. For that reason, we appreciate the opportunity to clarify and reinforce the principal scientific arguments presented in our paper.
